# Integrative Analysis of ATAC-Seq and RNA-Seq Identifies Key Genes Affecting Muscle Development in Ningxiang Pigs

**DOI:** 10.3390/ijms26062634

**Published:** 2025-03-14

**Authors:** Wenhua Tan, Chenxi Liu, Juan Liu, Sheng Wen, Yantong Chen, Ruimin Ren, Ning Gao, Xiaoling Ding, Jun He, Yuebo Zhang

**Affiliations:** 1 Key Laboratory of Livestock and Poultry Resources (Pig) Evaluation and Utilization, Ministry of Agriculture and Rural Affairs, College of Animal Science and Technology, Hunan Agricultural University, Changsha 410128, China; 1353933404@stu.hunau.edu.cn (W.T.); liuchenxi0018@hunau.edu.cn (C.L.); liujuanid@hunau.edu.cn (J.L.); 15885463786@stu.hunau.edu.cn (S.W.); chenyt0298@163.com (Y.C.); ruimin.ren@hunau.edu.cn (R.R.); gaon@hunau.edu.cn (N.G.); 2 Yuelushan Laboratory, Changsha 410128, China; 3 College of Animal Science and Technology, Anhui Agricultural University, Hefei 230036, China; dinxiaoling1997@ahau.edu.cn

**Keywords:** Ningxiang pigs, muscle development, ATAC-seq, RNA-seq, chromatin accessibility

## Abstract

Meat production traits in pigs are critical economic characteristics, primarily influenced by the formation and development of skeletal muscle. Skeletal muscle development is regulated by a complex transcriptional network, which partly relies on chromatin accessibility for initiation. Ningxiang pigs, a renowned Chinese indigenous breed, are highly valued for their tender meat. However, studies focusing on skeletal muscle development in Ningxiang pigs, particularly from the perspective of chromatin accessibility, have not yet been reported. Based on this, the present study selected several key time points in the skeletal muscle development of Ningxiang pigs to perform Transposase-Accessible Chromatin Sequencing (ATAC-seq) and RNA sequencing (RNA-seq). This was carried out to identify key open chromatin regions and genes during different growth stages, which could influence skeletal muscle development in Ningxiang pigs. We collected longissimus dorsi muscle samples at postnatal days 14 (D14), 28 (D28), 85 (D85), 165 (D165), and 250 (D250). For each age, three individuals were collected for ATAC-seq and RNA-seq. After initial differential analysis among different ages, we identified 6412 differentially accessible chromatin peaks and 1464 differentially expressed genes. To clarify the key candidate transcription factors affecting the development of skeletal muscle in Ningxiang pigs, motif analysis of differential peaks revealed potential cis-regulatory elements with binding sites for transcription factors, including *Fosl2* and *JunB*. Correlation analysis identified 56 overlapping genes and a significant positive correlation (r = 0.73, *p* = 1 × 10^−14^) between gene expression and chromatin accessibility. Key candidate genes such as *HOXA10*, closely related to skeletal muscle development, were specifically examined. These results enhance our understanding of the genetic and epigenetic regulatory mechanisms of porcine skeletal muscle development, providing a robust foundation for future molecular studies.

## 1. Introduction

Pork is one of the primary sources of protein in the human diet and is also an indispensable model in biomedical research [1,2]. Ningxiang pig is renowned for its tender and flavorful meat, as well as its relatively high intramuscular fat content [3]. However, compared to foreign imported lean-type pig breeds, Ningxiang pigs have a slower growth rate and a lower lean meat percentage, which significantly limits their production efficiency [4]. Enhancing the growth rate and meat yield efficiency of indigenous pig breeds has been a longstanding focus of genetic breeding research. Thus, studying the molecular mechanisms driving Ningxiang pig skeletal muscle development is extremely significant.

The growth and development of skeletal muscle are highly complex and dynamic regulatory processes, in which the dynamic expression and interaction of genes and transcription factors at different stages are crucial for shaping the phenotype of the skeletal muscle [5]. Previous studies have shown that the expression of certain genes can influence skeletal muscle phenotypes. *RYR1* and *RN* have a significant impact on the composition of muscle fiber types and fiber size. Mutations in the *RYR1* gene have been found to increase the percentage of glycolytic muscle fibers (MyHC IIb) and the cross-sectional area of muscle fibers [6]. *PAX3* and *PAX7*, as key members of the transcription factor network, also play an important role in skeletal muscle development [7]. Also, basic helix–loop–helix (bHLH) myogenic regulatory factors (MRFs), such as *MyoD*, *Myf5*, *Myf6* (*MRF4*), and *MyoG*, play a crucial role in muscle development through their sequential expression [8]. However, studies focusing on the epigenetic regulation of pig skeletal muscle development are still limited, especially those on chromatin accessibility.

In the field of epigenetic regulation, the open state of chromatin in mammals is one of the key regulatory factors. In eukaryotic organisms, nuclear DNA is tightly wrapped around nucleosomes, with each nucleosome composed of a DNA wound around a histone octamer core. The interaction between transcription factors and DNA, as well as transcriptional regulatory functions, can lead to the dissociation of nucleosomes, thereby forming open chromatin regions [9]. Transcription factors (TFs) and other regulatory factors can only access chromatin regions and activate gene expression when these regions are in an open state. Conversely, when chromatin regions are closed, the binding of these transcription factors to regulatory regions is hindered, leading to gene silencing [10]. Currently, the Assay for Transposase-Accessible Chromatin Sequencing (ATAC-seq) has become the preferred method for studying chromatin structure [11]. Some researchers have already integrated ATAC-seq and RNA-seq for in-depth exploration of the skeletal muscle regulation mechanism of cattle and chickens [12,13,14]. In addition, studies have provided high-resolution landscapes of the transcriptome and chromatin accessibility of the pectoralis major muscle in broiler chickens and identified genes that play a key role in the post-hatch development of broilers, including *ACTC1*, *FDPS*, *NRG1*, *TGFB3*, *MUSTN1*, and *FOS* [15]. We aim to uncover the epigenetic regulatory mechanisms of postnatal skeletal muscle development in Ningxiang pigs, particularly how chromatin accessibility and transcriptomic dynamics jointly regulate the molecular network of muscle development.

In this study, we utilized ATAC-seq to quantify chromatin accessibility across various postnatal stages in the longissimus dorsi muscle of Ningxiang pigs, to identify transcription factors that regulate muscle growth and development. By integrating the analysis with RNA-seq data, we further determined several candidate core genes that may regulate muscle growth and development. These results will offer a valuable resource for further exploring the potential mechanisms of skeletal muscle growth and development.

## 2. Results

### 2.1. Morphological Characteristics of Muscle Fibers in Ningxiang Pigs

**Figure 1:**
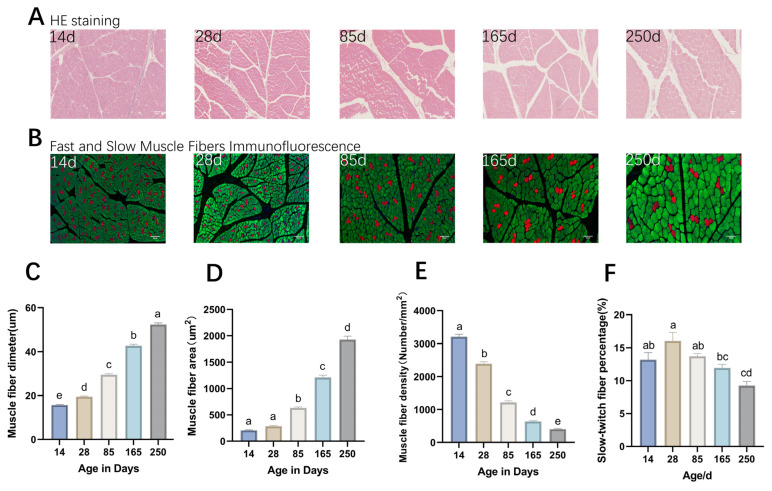
Morphological analysis of Ningxiang pig dorsal muscle tissue. (**A**) H&E (hematoxylin–eosin) staining results. (**B**) Results of immunofluorescence double staining of fast-twitch and slow-twitch muscle fibers, in which the fibers dyed red and green are slow-twitch muscle and fast-twitch muscle, respectively. (**C**–**F**) Histograms showing significant differences in muscle fiber diameter, area, density, and the proportion of slow muscle fibers at different developmental stages, respectively. Same letters in the graphs indicate non-significant differences, and different letters indicate significant differences (*p* < 0.05).

The analysis of muscle fiber-related indicators revealed that the diameter and area of muscle fibers in Ningxiang pigs increased significantly with age, with average daily increases of 0.27, 0.18, 0.16, and 0.11 µm and 5.50, 6.13, 7.24, and 8.45 µm^2^, respectively. It was observed that the increase in muscle fiber diameter slowed down progressively, while that in muscle fiber area accelerated over time (Figure 1C,D). The density of muscle fibers showed a decreasing trend with age, with average daily decreases of 58.21, 20.70, 7.19, and 2.75 fibers/mm^2^ (Figure 1E). The proportion of slow-twitch muscle fibers exhibited a fluctuating pattern across different ages, peaking at 16.02% at 28 days, which was significantly higher than that at 165 days (11.95%) and 250 days (9.26%) (*p* < 0.05) (Figure 1F).

### 2.2. Identification of Gene Expression Patterns in the Longissimus Dorsi Muscle of Ningxiang Pigs

**Figure 2:**
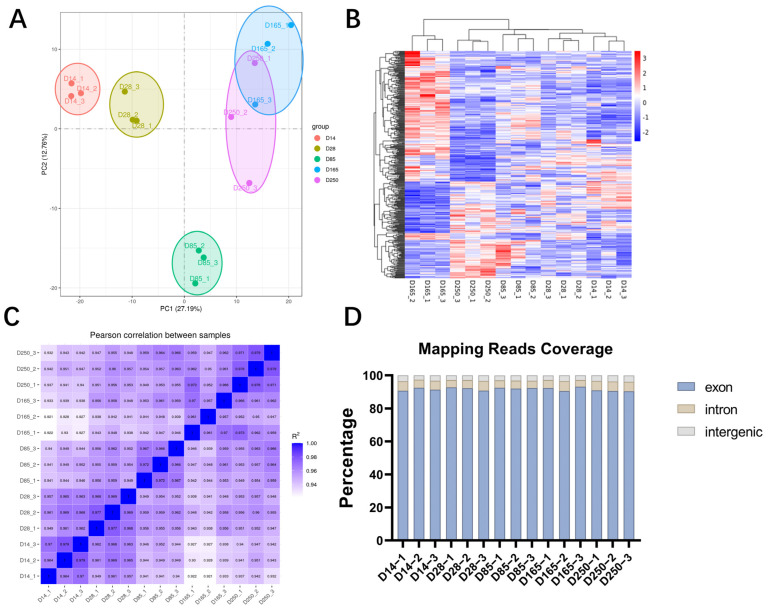
Summary of RNA-seq quality. (**A**) Principal component analysis (PCA) plot. (**B**) Heatmap of the distribution of gene expression levels in each sample. (**C**) Heatmap showing the Pearson correlation of overall gene expression levels between the samples. (**D**) The distribution of unique aligned sequences in each region of the reference genome.

To determine the gene expression patterns of Ningxiang pigs at different developmental stages, we selected samples from three castrated male pigs at each stage for RNA-seq. A total of 688,340,436 raw reads were obtained. After trimming and filtering, 666,309,246 clean reads were retained. Uniquely mapped rates ranged from 86.26% to 88.97% (Table S1). The principal component analysis (PCA) and heatmap indicated that the expression patterns were highly consistent within groups but distinct between groups (Figure 2A,B). The Pearson correlation coefficient was greater than 0.92 among samples with similar genetic backgrounds, which further validated the rationality of the sample classification (Figure 2C). The number of sequences aligned with exons, introns, and intergenic regions was counted, and the majority of sequences was localized to the exon regions (Figure 2D). This indicates that the genomic annotation data we used have a high degree of accuracy. In summary, the results indicate that the generated RNA-seq data are reliable.

### 2.3. Differential Expression Genes and Enrichment Analysis

**Figure 3:**
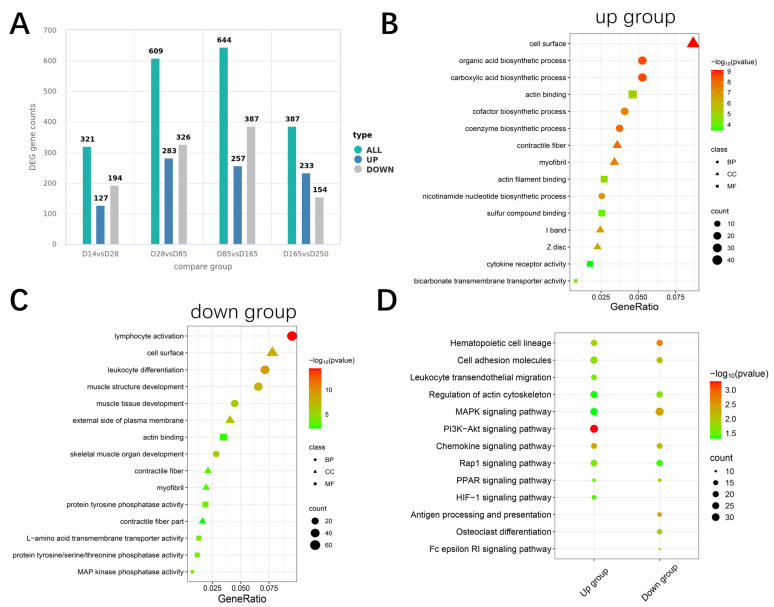
Enrichment analysis of differentially expressed genes. (**A**) Statistical plot of the number of significantly differentially expressed genes (DEGs) between groups. (**B**) Top 5 Gene Ontology (GO) terms enriched by upregulated DEGs across Biological Process (BP), Cellular Component (CC), and Molecular Function (MF). (**C**) Top 5 GO terms enriched by downregulated DEGs across BP, CC, and MF. (**D**) Top 10 KEGG entries enriched by up- and downregulated DEGs.

Comparisons between each group yielded 321, 609, 644, and 387 expressed genes (DEGs), respectively. The combined set from all comparisons included 900 upregulated and 1061 downregulated DEGs (Figure 3A). Functional enrichment analysis was performed on these DEGs for upregulated and downregulated DEGs. The upregulated DEGs were particularly enriched in biological synthesis, contractile fibers, and actin binding (Figure 3B). In contrast, downregulated genes were enriched in immune processes and muscle tissue structure development (Figure 3C). Kyoto Encyclopedia of Genes and Genomes (KEGG) pathway analysis revealed significant enrichment of multiple pathways related to energy metabolism and cell signaling, such as the MAPK signaling pathway, PI3K-Akt signaling pathway, Rap1 signaling pathway, PPAR signaling pathway, and HIF-1 signaling pathway, and regulation of the actin cytoskeleton (Figure 3D). Notably, seven pathways, including the hematopoietic cell lineage, regulation of the actin cytoskeleton, MAPK signaling pathway, and PPAR signaling pathway, were significantly enriched by both upregulated and downregulated genes. These findings suggest that DEGs may play crucial roles in muscle growth and development processes [16,17,18,19].

### 2.4. Trend Analysis of All Differentially Expressed Genes

**Figure 4:**
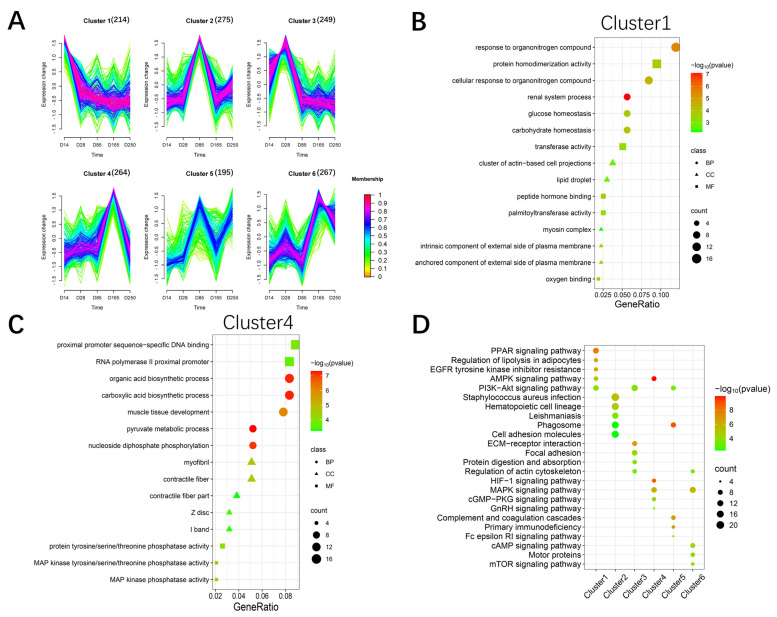
Cluster analysis for all RNA−seq genes. (**A**) Clustering of the genes based on their expression patterns at D14, D28, D85, D165, and D250. The numbers in parentheses indicate the number of genes in a cluster. (**B**) The significant GO terms of the gene set Cluster 1. (**C**) The significant GO terms of the gene set Cluster 4. (**D**) The significant KEGG pathways of the six clusters of genes.

We performed trend analysis using the Mfuzz package to obtain six gene expression trends across the five developmental stages of Ningxiang pigs (Figure 4A). GO enrichment analysis was conducted on the six clusters. Cluster 1 showed a continuously decreasing trend, with functions primarily enriched in organic nitrogen compound binding and actin-related processes (Figure 4B). The expression of the genes in Clusters 3 and 4 exhibited a trend of increasing first and then decreasing, with these genes mainly involved in cell proliferation, muscle tissue development, and organic acid biosynthetic processes (Figure 4C and Figure S1). The gene expression trends in Clusters 2 and 5 both showed an increase followed by a decrease and then another increase, and these genes are primarily associated with immune effector processes and cell activation (Figures S2 and S3). Genes in Cluster 6 were mainly enriched in biosynthetic processes and muscle structure development (Figure S4). KEGG enrichment analysis was performed to identify significant pathway maps across the six trend clusters. The pathways that were significantly enriched included the MAPK, AMPK, PPAR, PI3K-Akt, and HIF-1 signaling pathways (Figure 4D). These pathways have all been reported to be associated with the development of skeletal muscle [19,20,21,22,23]. Overall, the trend analysis revealed that gene functions were primarily enriched in biosynthesis, muscle tissue development, cell proliferation, and immune responses.

### 2.5. Validation of DEG Expression

**Figure 5:**
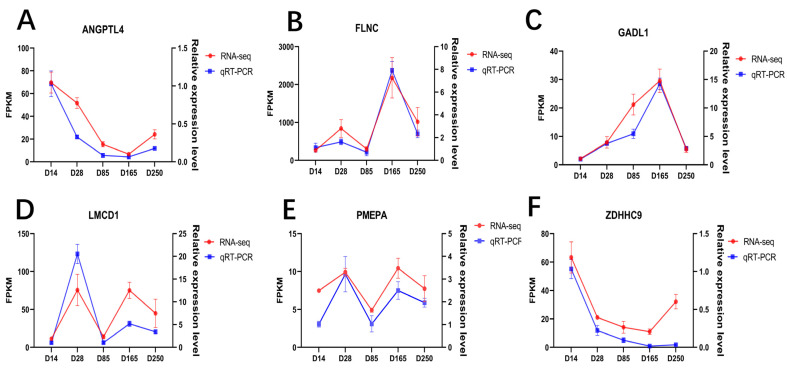
Quantitative validation of the RNA-seq data. Line charts of the qRT-PCR and RNA-seq data for the following genes: (**A**) *ANGPTL4* gene; (**B**) *FLNC* gene; (**C**) *GADL1* gene; (**D**) *LMCD1* gene; (**E**) *PMEPA* gene; (**F**) *ZDHHC9* gene.

To substantiate the accuracy of the transcriptomic data, we randomly selected six genes for qRT-PCR validation, including *ANGPTL4*, *FLNC*, *GADL1*, *LMCD1*, *PMEPA*, and *ZDHHC9*. The results revealed that the expression patterns obtained from RT-qPCR are consistent with those from RNA-seq, affirming the reliability of the DEGs identified in this study (Figure 5A–F).

### 2.6. Identification of Chromatin Accessibility Patterns in the Longissimus Dorsi Muscle of Ningxiang Pigs

**Figure 6:**
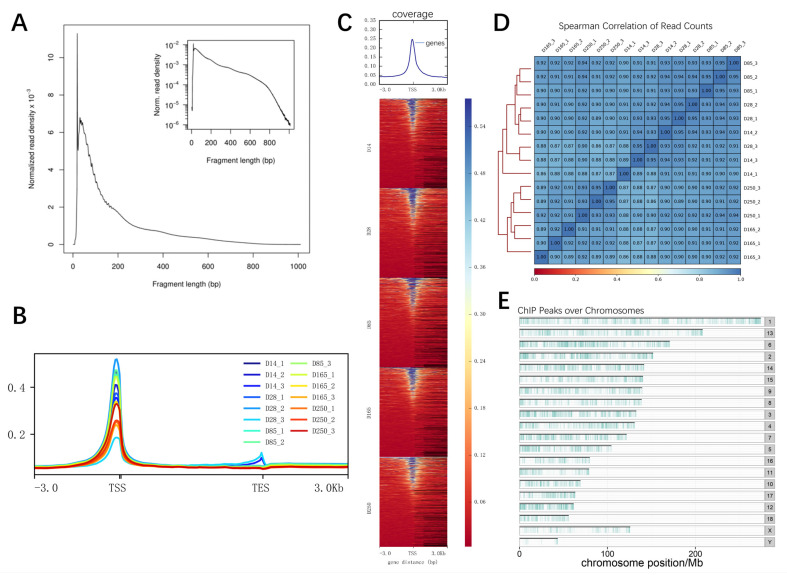
ATAC-seq data statistics. (**A**) Fragment length distribution map. (**B**) Distribution of mapped reads across gene bodies and peaks. (**C**) Heatmap of read distribution across gene bodies and peaks. The X axis represents the normalized gene or peak length, and the Y axis represents the read enrichment. TSS represents transcription start site. (**D**) Spearman’s correlation coefficients displaying the relationships between various samples presented in a heatmap scatterplot. (**E**) Distribution of peaks across the chromosomes.

To elucidate the chromatin accessibility regions involved in muscle development across the entire genome, an in-depth analysis utilizing ATAC-seq was conducted. A total of 1,332,874,079 raw reads were obtained, and after filtration, 1,306,701,560 clean reads were acquired, with an average of 91.34% of reads successfully mapped. The ATAC-seq samples all exceeded 70 million effective reads (Table S2). Initially, the quality of the libraries was assessed based on insert length and peak signal distribution. All libraries displayed the expected distribution of fragment lengths, including nucleosome-free fragments and mononucleosome fragments (Figure 6A). The majority of the identified accessible chromatin regions were concentrated within a 3kb region around the transcription start site (TSS), highlighting the importance of open chromatin regions in transcriptional regulation (Figure 6B,C). Spearman’s correlation coefficient, calculated based on the read signals, revealed variations across different ages. Coefficients closer to 1 indicate higher similarity in expression patterns between the two samples (Figure 6D). These results suggest that our sequencing data are of high quality. On average, 60,525 accessible chromatin peaks were identified per sample, and the distribution of peaks across the genome is illustrated in Figure 6E. It can be observed that accessible regions are widely distributed across the entire genome.

### 2.7. Enrichment Analysis of the Gene Annotations in Differentially Accessible Chromatin Regions

**Figure 7:**
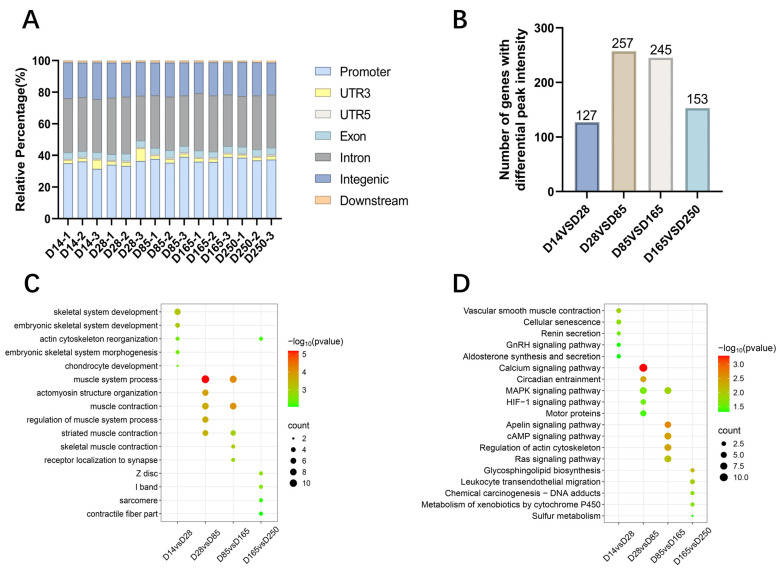
Functional annotation and enrichment of differential peaks. (**A**) Genomic distribution of accessible chromatin regions across muscle samples analyzed. (**B**) Gene annotation with differential chromatin accessibility regions identified between developmental stages D14 and D28, D28 and D85, D85 and D165, and D165 and D250. (**C**) GO enrichment categories for genes annotated with differential peaks. (**D**) KEGG enrichment pathways for genes annotated with differential peaks.

The annotation of open chromatin peaks across all samples revealed that the majority of these peaks were predominantly mapped to promoters, introns, and intergenic regions (Figure 7A). Subsequently, we identified differential peaks between each pair of developmental stages and annotated the genes located within a 3 kb region of these differential peaks. The results showed that during the myogenic transitions from D0 to D60, D28 to D85, D85 to D165, and D165 to D250, the differential peaks corresponded to 127, 257, 245, and 153 genes (Figure 7B), respectively. GO and KEGG pathway enrichment analyses were performed on these genes. The GO analysis revealed significant enrichment in biological processes such as skeletal system development, muscle contraction, and actin filament binding. The most significant terms are highlighted in Figure 7C. KEGG enrichment analysis identified a varying number of significant KEGG entries within each group, and some muscle development-related pathways were enriched, such as the GnRH, MAPK, HIF-1, Ras, Apelin, and cAMP signaling pathways (Figure 7D).

### 2.8. Motif Analysis of Differentially Accessible Chromatin Regions During Skeletal Muscle Development

**Figure 8:**
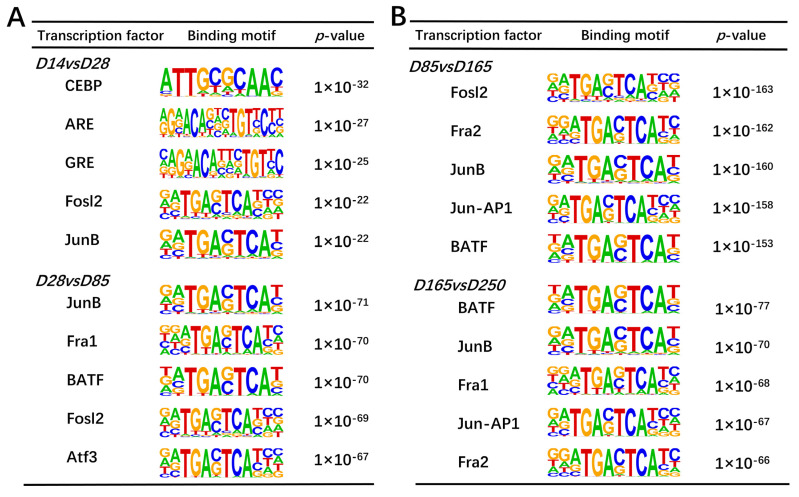
Motif enrichment analysis of differential peaks. (**A**) Top five transcription factor binding motifs enriched in significantly peak regions according to the *p*-values between D14 and D28 and between D28 and D85, respectively. (**B**) Top five transcription factor binding motifs enriched in significant peak regions according to the *p*-values between D85 and D165 and between D165 and D250, respectively.

We use the peak-calling function of the HOMER suite to detect open chromatin regions that may contain TF binding sites. By computationally deducing motifs within identified differential peak sequences, we can ascertain the TFs that may potentially bind to open chromatin regions under specific conditions. We have successfully identified 141, 139, 194, and 134 transcription factors with statistical significance (*p* < 0.01), respectively. Based on the *p*-values in the comparisons of D14 versus D28, D28 versus D85, D85 versus D165, and D165 versus D250, the top five transcription factor binding motifs were identified (Figure 8A,B). These motifs were enriched in significant peak regions, indicating their potential role in regulating skeletal muscle development. Among them, *JunB*, *Fosl2*, *Fra1*, and *BATF* were significantly enriched and were therefore considered as key regulatory factors.

### 2.9. Correlation Between Chromatin Accessibility and Gene Expression in Skeletal Muscle Tissue

**Figure 9:**
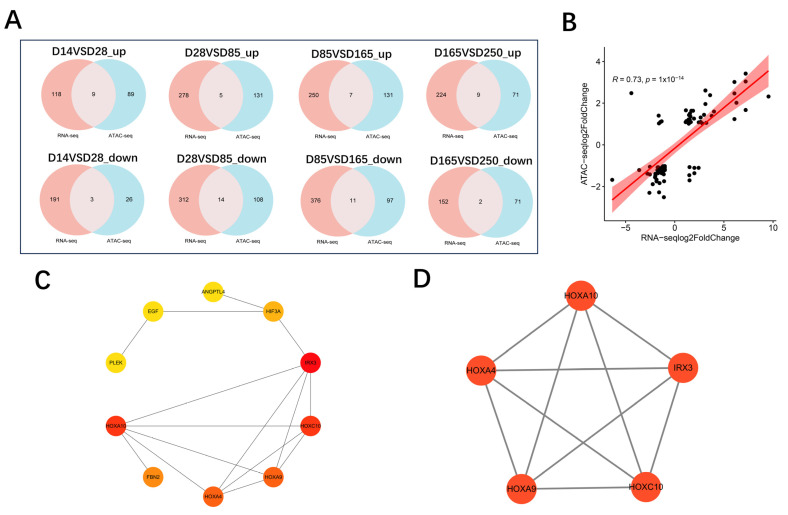
Integrated analysis for ATAC-seq and RNA-seq. (**A**) Overlap of differently expressed genes identified by RNA-seq and ATAC-seq. “Up” refers to upregulated expression and chromatin accessibility. “Down” refers to downregulated expression and chromatin accessibility. (**B**) Correlation analysis between chromatin accessibility and gene expression levels of overlapping genes. (**C**) Subnetwork of the top 10 hub genes screened by the MCC algorithm. (**D**) MCODE top module clustering.

To explore the relationship between open chromatin regions and gene expression, we performed a correlation analysis using ATAC-seq and RNA-seq data. By correlating the expression levels with chromatin openness across all overlapping genes, a significant positive correlation was revealed (r = 0.73, *p* = 1 × 10^−14^) (Figure 9B). Initially, in the comparisons of D14 vs. D28, D28 vs. D85, D85 vs. D165, and D165 vs. D250, the number of overlapping genes between significantly upregulated expression genes and the genes located within significantly upregulated accessible peaks was 9, 5, 7, and 9, respectively. Furthermore, the number of overlapping genes between significantly downregulated expression genes and the genes located within significantly downregulated accessible peaks was 3, 14, 11, and 2, respectively (Figure 9A). A total of 56 genes with consistent trends in both expression and chromatin accessibility were screened. HOXA10 exhibits highly significant differential expression and differential chromatin openness across multiple stages, and in conjunction with existing literature reports, we speculate that HOXA10 may be key candidate genes regulating the growth and development of pig skeletal muscle. To further identify hub genes, we utilized STRING to construct a Protein–Protein Interaction (PPI) network for the 56 overlapping genes (Figure S7). An integrated analysis was conducted on the genes obtained from both the MCC and MCODE algorithms (Figure 9C,D), ultimately leading to the identification of five core genes: *HOXA10*, *HOXA4*, *HOXA9*, *HOXC10*, and *IRX3*. We subsequently employed IGV (version 2.18.4) software to visualize the relationship between gene chromatin openness and gene expression. The candidate gene *HOXA10* exhibited an initial decrease in chromatin accessibility, followed by an increase and then a decrease again, with its transcription levels showing a consistent trend (Figure S5). In addition, the candidate gene *HOXA4* also exhibited a consistent trend in both transcription levels and chromatin accessibility, characterized by an initial decrease followed by an increase (Figure S6).

### 2.10. Protein–Protein Interaction Network Analysis

**Figure 10:**
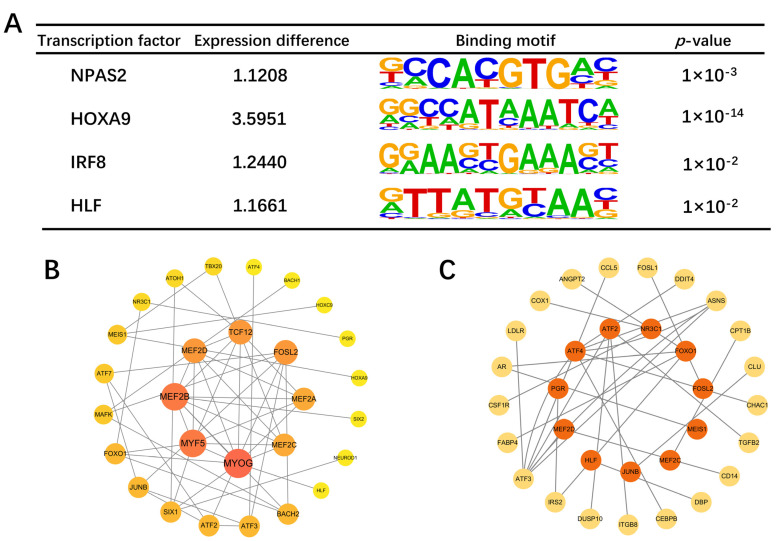
The PPI network of overlapping genes and TFs. (**A**) Four differentially expressed transcription factors. (**B**) TF interaction network. Node size and color indicate the number of connections to other TFs. (**C**) Network map of differential target genes regulated by transcription factors. The central orange ones are TFs, and the peripheral yellow ones are target genes.

The top 30 TFs from each group, identified through motif analysis, were used to construct an interaction network. *MYOG*, *MYF5*, *MEF2B*, *MEF2D*, *TCD12*, and *FOSL2* were identified as central TFs with the closest connections to other TFs (Figure 10B). Based on the TRRUST database, we compiled our TF–regulatory target gene relationships, encompassing 24 TFs and 195 target genes. Upon comparison of these target genes with DEGs, 21 overlapping genes were obtained (Figure S8), corresponding to 12 TFs (Figure 10C), many of which are associated with the growth and development of skeletal muscle, such as *ATF3* and *IRS2* [24,25]. To identify differentially expressed TFs, we used RNA-seq data and found four TFs to be differentially expressed: *NPAS2*, *HOXA9*, *IRF8*, and *HLF* (Figure 10A).

## 3. Discussion

The lean meat ratio of pigs is a crucial economic indicator, influenced by the growth and development of pig skeletal muscle [26]. This study initially obtained a profile of muscle fiber phenotypes at different postnatal developmental stages of Ningxiang pigs. The diameter and area of muscle fibers significantly increased with age, and the ratio of fast to slow muscle fibers fluctuated as development progressed, with a significant decrease in the proportion of slow muscle fibers in the later stages of growth. The muscle fiber density significantly decreased with age, which is consistent with previous research [27]. In recent years, research has focused on the transcriptome and proteome of skeletal muscle, as well as methylation in Ningxiang pigs [3,28]. However, there is a scarcity of studies from the perspective of chromatin accessibility. Therefore, we utilized ATAC-seq analysis to investigate chromatin accessibility differences in skeletal muscle growth and development across various growth stages of Ningxiang pigs. Furthermore, we integrated RNA-Seq data to identify potential key factors that may affect muscle development.

Initially, we identified the differentially expressed genes among the developmental stages D14, D28, D85, D165, and D250; this was followed by GO and KEGG enrichment analyses of these genes. Signaling pathways such as MAPK, AMPK, PI3K-Akt, and PPAR were identified as crucial for porcine skeletal muscle development [19,20,29]. It was also found that HIF-1 is related to muscle fiber types. HIF-1α plays a crucial role in regulating the glycolysis pathway in skeletal muscle. Its absence leads to a shift from glycolysis to oxidation during exercise, increasing exercise duration but also causing muscle damage [30]. In the ATAC-seq analysis of Ningxiang pig skeletal muscle, open chromatin regions were predominantly enriched in promoter regions and intronic regions, followed by intergenic regions. This pattern is expected due to the large genomic expanse of intergenic regions, which increases the likelihood of detecting open chromatin peaks. The significant enrichment of ATAC-seq peaks in promoter regions highlights the critical role of chromatin accessibility in transcriptional regulation. The average number of peaks across different developmental stages (D14, D28, D85, D165, and D250) in Ningxiang pig skeletal muscle were 66,889, 65,901, 54,579, 56,754, and 58,497, respectively. The time point with the highest number of open chromatin regions was 14 days postnatal, followed by 28 days, revealing that the regulatory genes of skeletal muscle are the most active during the early stages. These findings are consistent with previous research. The skeletal muscle of the Landrace pigs had the most peaks at 0 days [14,31]. A comparison analysis between different stages identified 1151, 1607, 2157, and 1498 differential peaks, respectively. Functional enrichment analysis of the genes located within these differential peaks revealed that they are primarily enriched in biological processes such as skeletal system development and muscle contraction, actin filament structure, and signaling pathways closely related to muscle development, such as the cAMP and MAPK signaling pathways. Previous studies have demonstrated that the cAMP signaling pathway plays a role in regulating muscle fiber size and metabolic phenotype, with its sustained activation leading to a significant hypertrophic response in skeletal muscle fibers through a series of complex molecular mechanisms [32]. Muscle fiber phenotype is regulated by several independent signaling pathways, including the mitogen-activated protein kinase (MAPK), nuclear factor of activated T cells (NFAT), myocyte enhancer factor 2 (MEF2), and peroxisome proliferator-activated receptor (PPAR) signaling pathways [33]. Interestingly, the enrichment analysis showed similar results for the differential genes observed in the RNA-seq analysis. Among the predicted transcription factor binding motifs, several key transcription factors related to muscle growth exhibited notable changes within peaks, which may reflect their activity in the regulation of muscle development. Key examples include *JunB* and *Fosl2*. Previous research has demonstrated that reducing the expression of *JunB* in adult skeletal muscle through RNA interference leads to atrophy. Moreover, the overexpression of *JunB* can induce hypertrophy without affecting satellite cell proliferation and independently stimulates protein synthesis, separate from the Akt/mTOR pathway. When *JunB* is transfected into denervated muscle, it can prevent fiber atrophy [34]. The significant inhibition of myogenic differentiation capacity by *Fosl2* knockdown highlights its central role in the H3K27ac-mediated regulatory network of muscle differentiation [35]. We also found four TFs to be differentially expressed (*NPAS2*, *HOXA9*, *IRF8,* and *HLF*). *NPAS2* regulates genes like *MYOD* and *MYOG*, which are key factors in myoblast differentiation. In vitro experiments indicate that *NPAS2* may control the timing of muscle differentiation by influencing the rhythmic expression of these genes [36], and *HLF* has been identified as a transcription factor that may play a key regulatory role in the fate transition of muscle cells [37].

We integrated ATAC-seq and RNA-seq data to further explore the potential correlation between chromatin accessibility and gene expression levels. A total of 56 overlapping genes were identified, showing co-expression in both ATAC-seq and RNA-seq datasets. When comparing gene expression and chromatin openness across multiple developmental stages, *HOXA10* genes demonstrated significant differences, and previous studies have reported their close association with muscle development. *HOXA10* inactivation led to genomic instability and mitotic catastrophe in somite-derived satellite cells in mice and humans. Satellite cell-specific *HOXA10* ablation in mice resulted in a decline in the regenerative ability of somite-derived muscles [38]. In muscle stem cell (MuSC)-specific *HOXA10* knockout mice, limb muscle regenerative capacity was significantly impaired, indicating that *HOXA10* is critical for maintaining region-specific muscle repair. In a Duchenne muscular dystrophy (DMD) model (mdx mice), *HOXA10* deficiency resulted in systemic muscle atrophy (except in the head). Notably, the expression patterns and functional roles of *HOXA10* in mouse and human limb MuSCs are highly conserved, which suggests its evolutionarily conserved role in mammalian muscle regeneration [39]. These findings suggest that the *HOXA10* genes may be significant target genes for pig skeletal muscle development in our study. Additionally, in the comparison of the two omics datasets, we observed a significant change in the number of emerging and disappearing peaks around the candidate key genes. We hypothesize that variations in gene expression result from differential binding of transcription factors to their regulatory sites, leading to precise regulation of gene activity. Lean meat percentage, as a crucial indicator of pork quality and economic value, is directly influenced by the development of skeletal muscle. In practical livestock production, optimizing skeletal muscle development through genetic selection and other means can significantly enhance lean meat percentage. Studies have shown that, compared with the fat-type Tongcheng pigs, the lean-type Landrace pigs exhibit stronger myoblast proliferation and cell migration efficiency during the embryonic period, which directly promotes an increase in muscle fiber numbers and thereby raises the proportion of muscle tissue [5].

Overall, our study provides a novel perspective on the dynamics of transcriptomic changes and chromatin accessibility during postnatal skeletal muscle development in Ningxiang pigs.

## 4. Materials and Methods

### 4.1. Sample Collection

The Ningxiang pigs used in this study were all procured from Hunan ChuWeiXiang Agriculture and Animal Husbandry Co Ltd. (Changsha, China). We selected pigs at various ages—14 (D14), 28 (D28), 85 (D85), 165 (D165), and 250 (D250)—with three castrated male pigs and three castrated female pigs from each age group for slaughter and sampling. All Ningxiang pigs were raised under identical environmental conditions and had free access to food. After slaughter, samples of the longissimus dorsi muscle were collected. A portion of the muscle was trimmed into 1 cm^3^ tissue blocks for morphological assessment of muscle fibers, while the remainder was stored in liquid nitrogen for RNA extraction and subsequent experiments. Six pigs at each age were used for the morphological analysis of muscle fibers and immunofluorescence staining. ATAC-seq and RNA-seq were conducted on samples from three castrated male pigs at each stage, unifying gender to reduce the impact on data. All Ningxiang pigs within the same time period are half-sibs. The comparative approach, which included time points such as D14 vs. D28, D28 vs. D85, D85 vs. D165, and D165 vs. D250, was used based on the phased and programmed nature of skeletal muscle development. This method allows for a clearer understanding of the sequential changes that occur during muscle development at different stages.

### 4.2. Morphological Analysis of Muscle Fibers

In this study, fresh muscle tissue samples were fixed in a 4% paraformaldehyde solution before paraffin embedding and sectioning. After solidification, the sections were stained with hematoxylin and eosin (H&E), sealed with neutral resin, and scanned using a digital slide scanner. Subsequently, CaseViewer (Version 2.4) image analysis software was used to randomly select at least 50 muscle fibers from each section. The diameters and areas of these fibers were manually measured, and the average values were calculated. Additionally, five randomly selected 1 mm^2^ fields from each section were used for manual counting of muscle fibers using ImageJ (Version 1.51) software. Muscle fiber density was determined by dividing the number of muscle fibers by the field area.

### 4.3. Immunofluorescence Staining

After dewaxing the paraffin sections and rehydrating them to water, antigen retrieval was performed. After the retrieval was completed, the slides were allowed to cool naturally and then washed in PBS (pH 7.4) three times, each for 5 min. Subsequently, endogenous peroxidase was blocked with 3% hydrogen peroxide, and the slides were incubated at room temperature in the dark for 25 min and washed with PBS three times. After blotting away the PBS, BSA and rabbit serum were added for blocking, and the slides were incubated for 30 min. The blocking solution was removed, the primary antibody was added, and the slides were incubated overnight at 4 °C. After washing, the HRP-labeled secondary antibody was added, and the slides were incubated at room temperature for 50 min. After washing, TSA dye was added, and the slides were incubated at room temperature in the dark for 10 min and washed with TBST three times. The sections were microwaved on medium heat for 8 min; the microwave was then stopped for 8 min and switched to medium–low heat for 7 min. The primary and secondary antibody incubation steps were repeated. DAPI staining was performed. After DAPI staining, the slides were incubated at room temperature in the dark for 10 min and then washed with PBS three times. Self-fluorescence quenching agent B was added, and the slides were incubated for 5 min and rinsed with running water for 10 min. Finally, anti-fade mounting medium was used to mount the slides, and the researcher captured images. The primary antibodies used were anti-fast myosin skeletal heavy chain (1:5000, GB112130, Servicebio, Wuhan, Hubei Province, China) and anti-slow myosin skeletal heavy chain (1:5000, GB111857, Servicebio). The secondary antibody was HRP-conjugated goat anti-rabbit IgG (1:500, GB23303, Servicebio).

### 4.4. RNA Sequencing

Total RNA was extracted from all skeletal muscle tissues using the traditional Trizol method. RNA integrity was assessed using the Agilent 2100 Bioanalyzer (Agilent Technologies, CA, USA). After RNA quality verification, NEB library construction was performed, and the libraries that passed quality control were sequenced on an Illumina platform, generating 150 bp paired-end reads. The raw reads were first filtered using fastp (Version v 0.20.0) software. Clean reads were then aligned to the reference genome (Sus scrofa 11.1) using HISAT2 (v2.0.5) [40,41]. The number of reads mapped to each gene was counted using FeatureCounts (v1.5.0-p3). FPKM (Fragments Per Kilobase of transcript per Million mapped reads) for each gene was calculated based on the gene’s length and the number of mapped reads. Differential expression analysis was performed using DESeq2 software (v1.20.0) [42,43]. *p*-values were adjusted using the Benjamini–Hochberg method to control the false discovery rate. Significant differentially expressed genes (DEGs) were identified based on the criteria of *padj* < 0.05 and |log2FoldChange| ≥ 1. Gene expression patterns were analyzed using the Mfuzz R package.

### 4.5. Quantitative Real-Time PCR (RT-qPCR)

Six differentially expressed genes (DEGs) were randomly selected for validation using quantitative real-time PCR (RT-qPCR). The RT-qPCR experiments were conducted using the All-In-One 5× RT MasterMix (Applied Biological Materials Inc., Richmond, BC, Canada). The expression levels of the target genes were calculated using the 2^−∆∆Ct^ method. The primers used in this study are shown in Table S3.

### 4.6. ATAC Sequencing

Nuclei were extracted from the samples and resuspended in a Tn5 transposase reaction mixture. The transposition reaction was incubated at 37 °C for 30 min. Following the transposition, equimolar amounts of Adapter1 and Adapter2 were added, and the library was amplified via PCR. After the PCR reaction, the library was purified using AMPure beads, and the quality was assessed with Qubit. Sequencing was performed on the Illumina Novaseq platform to generate 150 bp paired-end reads. Raw reads were initially processed with fastp (v 0.20.0) to obtain high-quality clean reads. Clean reads were aligned to the reference genome (*Sus scrofa* 11.1) using BWA (v 0.7.12), and reads derived from mitochondrial DNA were removed. The reads were then filtered to obtain high-quality ones (MAPQ ≥ 13), and those that did not properly pair and PCR duplicates were removed. Uniquely mapped and deduped reads were used for further analysis [44]. All peaks were called using MACS2 (v 2.2.7.1) with the command “MACS2-q 0.05--call-summits--nomodel--shift-100--extsize 200--keep-dup all”, defaulting to a *q*-value < 0.05 for peak identification. Peaks from different groups were merged using bedtools merge, and RPM values were calculated using bedtools coverage-count. The mean RPM of the biological replicates was computed, and the log2 fold change (log2fc) value was calculated as log2 (RPM of treatment group/RPM of control group). Only peaks with |log2fc| > 1 and *p*-value < 0.05 were considered as differential peaks [45,46]. The ChIPseeker (Version 1.41.0) software was utilized to identify genes associated with the peaks and to annotate the genomic regions of these peaks. Specifically, ChIPseeker assigned peaks to genes if they were located within ±3000 bp of a gene’s transcription start site (TSS) [47]. Motif analysis within differential peaks was performed using the findMotifsGenome.pl program in HOMER (v4.11).

### 4.7. Enrichment and Protein–Protein Interaction Network Analysis

Differentially expressed genes, along with genes annotated from differential peaks, were used for enrichment analysis of Gene Ontology (GO) and Kyoto Encyclopedia of Genes and Genomes (KEGG). GO and KEGG enrichment analysis were performed using clusterProfiler software (version 3.8.1) with the significance level of the corrected *p*-value set to < 0.05. Using the STRING database, a Protein–Protein Interaction (PPI) network was constructed. The top 10 hub genes were identified through the MCC algorithm (Maximal Clique Centrality). Subsequently, the MCODE algorithm was employed to identify significant clustering modules within the PPI network, and the top module was selected. The transcriptional regulatory relationships between TFs and target genes were obtained from the Transcriptional Regulatory Relationships Unraveled by Sentence-based Text Mining database (TRRUST; https://www.grnpedia.org/trrust/ (accessed on 31 December 2024)).

## 5. Conclusions

In this study, using RNA-seq analysis, we identified 1464 significantly differentially expressed genes, including key regulators such as *HOXA10*, which are closely associated with pig skeletal muscle development. Additionally, ATAC-seq analysis revealed the presence of potential cis-regulatory elements enriched with binding sites for various transcription factors, including *JunB* and *Fosl2*. Together, these analyses have enabled the construction of a map of gene expression and chromatin accessibility during the postnatal development of pig skeletal muscle. This comprehensive resource not only deepens our understanding of the molecular mechanisms underlying skeletal muscle development in pigs but also provides a valuable foundation for future genetic improvement efforts and practical applications in swine production systems.

## Data Availability

The raw sequence data reported in this paper have been deposited in the Genome Sequence Archive (Genomics, Proteomics & Bioinformatics 2021) in National Genomics Data Center (Nucleic Acids Res 2022), China National Center for Bioinformation/Beijing Institute of Genomics, Chinese Academy of Sciences (GSA: CRA021409; CRA021199), which are publicly accessible at https://ngdc.cncb.ac.cn/gsa (accessed on 14 December 2024) (accession numbers: PRJCA033677; PRJCA033527).

## Supplementary Materials

The supporting information can be downloaded at https://www.mdpi.com/article/10.3390/ijms26062634/s1.
