# Supplementary material for: Integrative Analysis of ATAC-Seq and RNA-Seq Identifies Key Genes Affecting Muscle Development in Ningxiang Pigs
Source: Int J Mol Sci. 2025 Mar 14;26(6):2634. doi: 10.3390/ijms26062634 (PMC11941884; doi:10.3390/ijms26062634)
Supplement: Supplementary file 1 [file ijms-26-02634-s001.zip › ijms-3482599-supplementary.pdf]

Table S1. RNA-seq data statistics.

Sample	Raw Reads (M)	Raw bases (G)	Clean reads (M)	Clean bases (G)	Q30 (%)	Unique mapped reads (%)
D14-1	43.30	6.49	42.09	6.31	95.15	88.91
D14-2	45.28	6.79	43.77	6.57	95.16	88.97
D14-3	49.93	7.49	48.60	7.29	94.56	87.84
D28-1	45.59	6.84	44.18	6.63	94.37	87.39
D28-2	48.38	7.26	46.82	7.02	94.56	86.98
D28-3	49.03	7.36	47.52	7.13	94.92	87.46
D85-1	48.71	7.31	46.99	7.05	94.79	87.83
D85-2	42.98	6.45	41.67	6.25	94.54	86.98
D85-3	47.55	7.13	45.95	6.89	95.05	87.94
D165-1	44.29	6.64	42.70	6.4	94.47	87.26
D165-2	48.31	7.25	46.43	6.96	94.53	87.2
D165-3	39.84	5.98	38.74	5.81	94.52	87.65
D250-1	49.71	7.46	48.30	7.24	94.48	87.88
D250-2	42.62	6.39	41.43	6.21	94.53	88.51
D250-3	42.80	6.42	41.11	6.17	93.8	87.93

Table S2.ATAC-seq data statistics.

Sample	Raw Reads (M)	Raw bases (G)	Clean reads (M)	Clean bases (G)	Q30 (%)	Unique mapped reads (%)
D14-1	93.18	27.95	91.63	19.34	91.76	92.12
D14-2	92.60	27.77	90.89	19.97	92.66	93.10
D14-3	97.97	29.39	95.31	21.17	92.10	86.23
D28-1	86.01	25.80	84.43	17.61	92.34	92.21
D28-2	85.07	25.52	83.92	16.41	93.00	92.51
D28-3	100.85	30.25	97.59	21.58	93.05	86.34
D85-1	73.36	22.01	72.06	15.37	93.00	92.41
D85-2	91.97	27.59	90.60	18.09	93.82	91.74
D85-3	76.96	23.09	75.57	16.18	92.30	92.10
D165-1	94.41	28.32	92.43	20.77	92.09	93.91
D165-2	96.48	28.94	94.18	21.46	91.59	92.18
D165-3	92.80	27.84	91.11	19.39	92.56	92.20
D250-1	74.79	22.44	73.90	13.06	92.67	90.04
D250-2	86.51	25.95	84.90	16.58	93.00	91.33
D250-3	89.95	26.99	88.16	17.73	92.63	91.67

Table S3. Information on primers used in this study.

Primer	Sequence (5’ -3’)
GAPDH-F	GTCGGAGTGAACGGATTTGGC
GAPDH-R	GGAGGTCAATGAAGGGGTCA
LMCD1	CCAGGAGTTCAGAAGATGTCCC
LMCD1	GAACACGTCCCCTTGCATCT
FLNC	GTTCCGTTGCACATACAGGC
FLNC	GTTCCGTTGCACATACAGGC
PMEPA1-F	CCAGAGCATGGAGATCACGGA
PMEPA1-R	CGAGCGGACAGCTTGTAGTG
ANGPTL4-F	GTAATTCAGAGGCGCCAGGA
ANGPTL4-R	CAGCCAGAATTCACCTTTGGG
GADL1-F	CTGGGCACATTAGGCCTTGAA
GADL1-R	ATTGGCATATTCAGGTTCCAGC
ZDHHC9-F	GTTTAGGGCCCGGACCATTG
ZDHHC9-R	AGGAGAGCTGGGCCTGAA

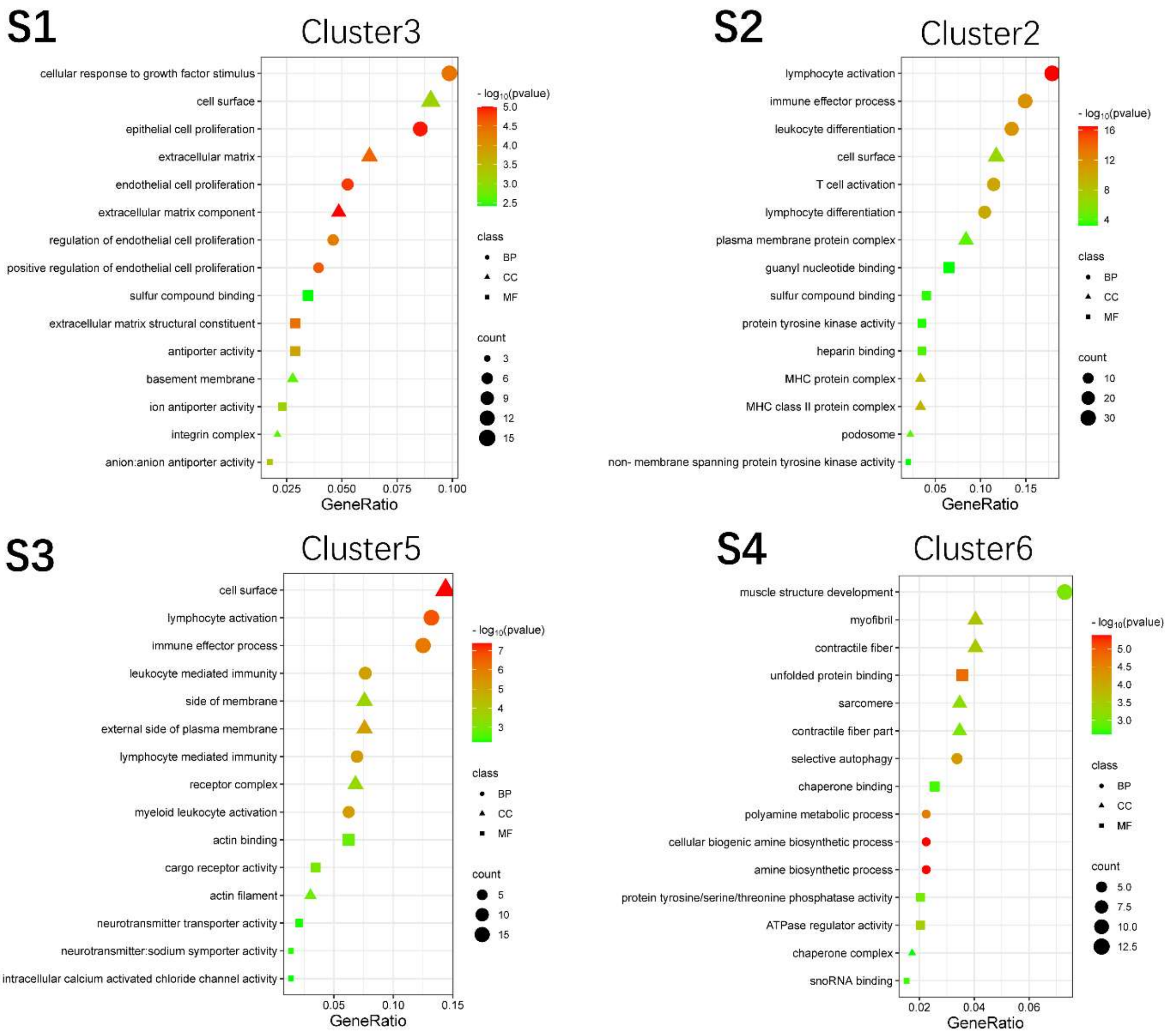

Figure S1、2、3、4. The biological process map of GO enrichment analysis after all genes in Cluster 3、2、5、6 was obtained after trend analysis during skeletal muscle development in NingXiang pigs.

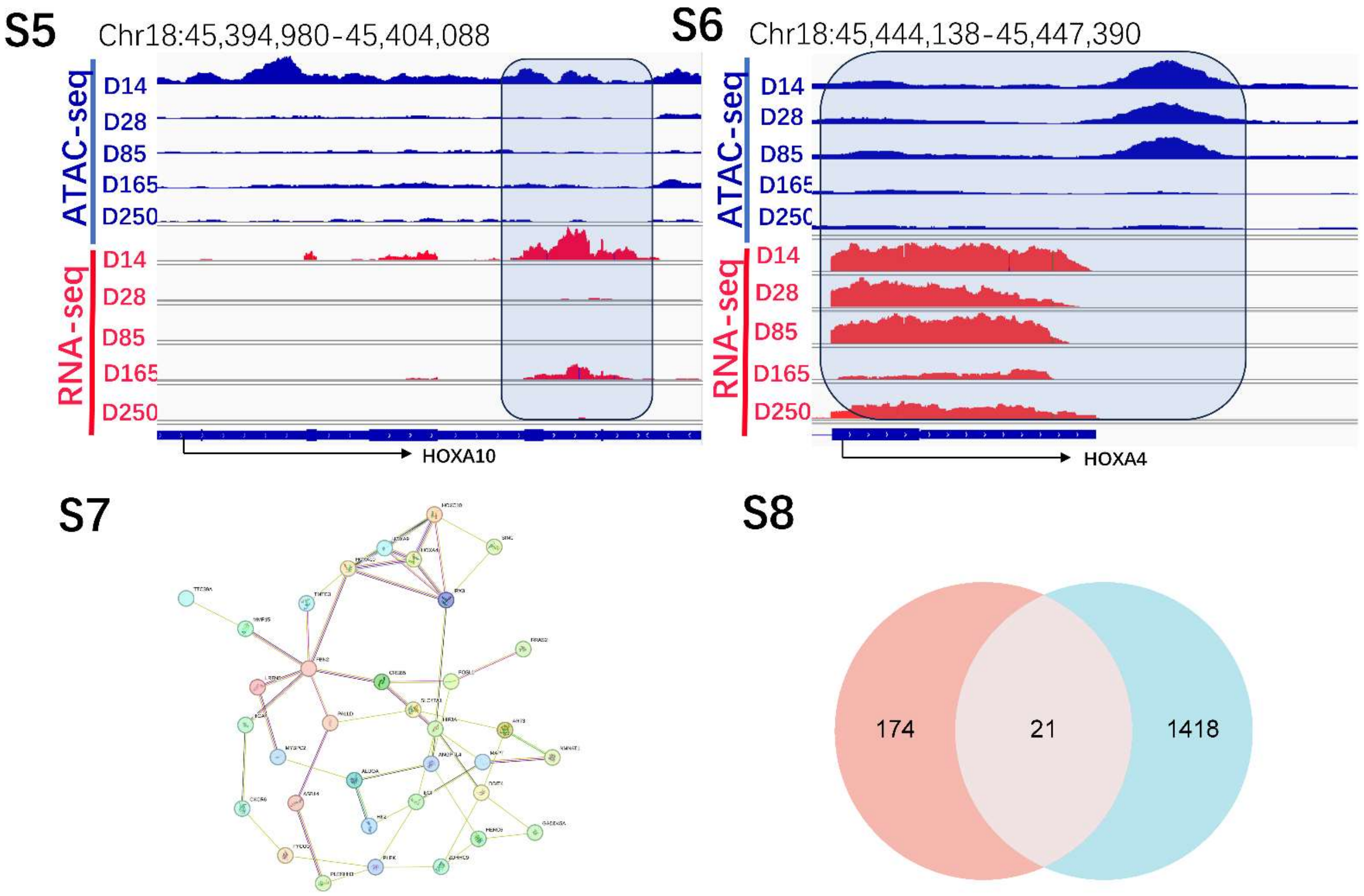

Figure S5. IGV plots showing chromatin accessibility and gene expression levels of the HOXA10 gene. **Figure S6.** IGV plots showing chromatin accessibility and gene expression lev-els of the HOXA4 gene. **Figure S7.** Overlapping gene interaction network. **Figure S8.** TFs target genes overlap with DEGs identified by RNA-seq.
